# Lecture on Eczema, Including Its Impetiginous, Lichenous, and Pruriginous Varieties

**Published:** 1863-10

**Authors:** T. McCall Anderson

**Affiliations:** Physician to the Dispensary for Skin Diseases; Physician to the Deaf and Dumb Institution, etc.


### LECTURE ON ECZEMA,

### INCLUDING ITS IMPETIGINOUS, LICHENOUS, AND PRURIGINOUS VARIETIES,

DELIVERED AT THE

DISPENSARY FOR SKIN DISEASES, GLASGOW, SCOTLAND.

By T. McCALL ANDERSON, M.D., F.F.P.S., Physician to the Dispensary for Skin Diseases; Physician to the Deaf and Dumb Institution, etc.

Reprinted from the "Medical Times and Gazette."

Gentlemen:—From the treatment of eczematous eruptions occurring in limited patches, I pass naturally to the consideration of the last division of the subject, the

*Local Varieties of Eczema,* and I trust you will bear in mind that the remarks I am about to make are to be taken in connexion with what I have already said, as much needless repetition may thus be avoided.

While eczema may be observed upon any part of the cutaneous envelope, and indeed may affect almost the whole of it at one time, there are certain localities which it seizes upon in preference to others, and to which it is often limited. These are the head, hairy portions of the face, lips, edges of the eyelids, nostrils, external auditory passages and ears, hands, feet, legs, genitals, anus, umbilicus, and those parts of the skin which are naturally in contact with one another.

*Eczema of the Head* (eczema capitis, impetigo capitis) occurs most frequently in the pustular form, especially in the case of children, whose heads are attacked with remarkable frequency. When this part is affected, the eruption has a tendency to chronicity, particularly if the treatment is not energetically and thoroughly carried into effect. For it is more difficult to keep the surface clean than when the non-hairy parts are invaded, owing to the hairs being glued together by the exudation, and to the crusts being entangled in them and difficult of removal. For this reason, the patient often allows them to remain for weeks, months, nay, even years upon the head, and, when advice is at last obtained, the whole scalp is not unfrequently found to be concealed from view. In this way, collections of pus are often formed between the crusts and the scalp, owing .to confinement of successive exudations, which do infinite harm. Besides, when hard crusts are allowed to remain on the head for a lengthened period of time, they press upon the hair follicles, and lead to their obliteration, whereas, when the eruption is properly treated from the first, there should be no permanent loss of hair. When the disease is neglected in the manner just indicated, the exudation becomes decomposed, lice are attracted to the part, and are often detected, wallowing in the mire, in thousands, while their nits (eggs) adhere, by means of sheaths, with great tenacity to the hairs, and in countless numbers. But while lice often occur as complications of an eczematous eruption, you must be alive to the fact, that these insects sometimes attack the head of a healthy person, in whom they excite a sensation of itching. This causes the patient to scratch the head, and an eczematous eruption may thereby be induced. The lice on the head are thus the exciting cause of eczema in some cases, its result in others.

Little sub-cutaneous abscesses are often met with on the head in addition to the collections of matter between the scalp and the crusts, and enlargement of the neighboring glands, especially those on the back of the neck and over the mastoid processes, occur in all aggravated chronic cases. These enlargements are due to the irritation set up by the adjacent eruption, and must on no account be looked upon as indicative of a strumous taint, though, of course, a smaller amount of irritation is capable of causing their enlargement in scrofulous children.

The *diagnosis* of eczema capitis is sometimes difficult to the unaccustomed eye, and I have accordingly arranged in a tabular form the points to be attended to as distinguishing it from syphilitic eczema capitis, seborrhœa capitis, psoriasis capitis, and herpes tonsurans.

*Table showing the Points which distinguish Eczema Capitis from Syphilitic Eczema Capitis, Seborrhœa Capitis, Psoriasis Capitis, and Herpes Tonsurans:—*

| Eczema Capitis. | Syphilitic Eczema Capitis. |
|---|---|
| (1.) Occurs oftenest in children. | (1.) Occurs usually in adults. |
| (2.) Often attacks the whole scalp. | (2.) Usually occurs in small patches. |
| (3.) Exhibits superficial ulcers only. | (3.) Exhibits deep ulcers, with perpendicular edges and unhealthy bases. |
| (4.) Occurs in persons in whom there is no history of primary syphilis, except as a coincidence. | (4.) Occurs in persons in whom there is a history of primary syphilis. |
| (5.) Does not occur in connexion with symptoms of syphilis, except as a coincidence. | (5.) Occurs in connexion with other signs of syphilis, *e.g.*, alopecia, sore throat, other syphilitic eruptions on the skin, glandular enlargements, rheumatic pains, etc. |

For further particulars, see the general diagnosis of eczema from syphilitic eruptions.

| Eczema Capitis. | Seborrhœa Capitis. |
|---|---|
| (1.) Exhibits crusts, which are brittle, are often very thick, and are composed of pus, granular matter, and epithelium. | (1.) Exhibits crusts, which can be kneaded into a ball, are usually thin, have an oily feel, and are composed principally of sebaceous matter and epithelium. |

| | |
|---|---|
| (2.) Is excessively itchy, and after removing the crusts, the scalp is infiltrated, red, often excoriated, and exudes serum or pus. | (2.) Is not excessively itchy, and, after removing the crusts, the scalp is not infiltrated, red, or excoriated, exudes neither serum or pus, but is smooth and oily. |

| ECZEMA CAPITIS. | PSORIASIS CAPITIS. |
|---|---|
| (1.) Occurs oftenest in those whose health is deteriorated, or who are scrofulous. | (1.) Occurs oftenest in those who are apparently in perfect health, and rarely, if ever, in those who are scrofulous. |
| (2.) Is usually very itchy. | (2.) Is usually not very itchy. |
| (3.) Exhibits exudation on the surface of the skin. | (3.) Is a perfectly dry eruption. |
| (4.) Exhibits thick yellowish, usually moist crusts. | (4.) Exhibits usually thin, white, dry scales. |
| (5.) Occurs often in connexion with eczema of other parts, as of the ears, etc. | (5.) Occurs almost invariably in connexion with psoriasis of other parts, especially of elbows and knees, where the diagnois is easy. |

| ECZEMA CAPITIS. | HERPES TONSURANS (Ringworm of the Head). |
|---|---|
| (1.) Patches not circular unless the hair has been cut short in a circular manner with scissors. | (1.) Patches circular. |
| (2.) Hairs healthy, (though they may fall out here and there,) and exhibit no parasite. | (2.) Hairs brittle, twisted, broken off close to the scalp, loaded with the parasite (tricophyton tonsurans). |
| (3.) Itching usually excessive. | (3.) Itching not usually severe. |
| (4.) Eczematous eruptions often on no other part of the body. | (4.) Herpes circinatus (ringworm of the body) often on the body. |
| (5.) Not contagious. | (5.) Contagious, especially to children, and often other members of the family exhibit ringworm of the head or body. |

But cases are often met with in which ringworn of the head is complicated with eczema of the head. The eczematous eruption is then the more prominent of the two, and the ringworm is often overlooked. In these cases, the diagnosis is arrived at by detecting some of the brittle, broken, or twisted hairs loaded with the parasite. It is, therefore, well, in every case of eczema, to examine the hairs carefully, with the eye at least. The history of the case, the way the eruption commenced, (in circular dry patches,) and the signs of the contagious nature of ringworm, assist the diagnosis. The following case is a good example of the complication of ringworm of the head with eczema:—

Richard R., aged 8, was admitted at the Dispensary for Skin Diseases, November 25, 1861. Almost the whole of his head was covered with a thick, yellowish, eczematous crusts, and the backs of his ears were infiltrated, exuding, and itching. Little patches of alopecia existed on the scalp, and on examining the head attentively, little fragments of hairs were detected here and there, which were brittle, broke on attempting to extract them entire, and were loaded with the spores of the tricophyton tonsurans. The disease commenced as a small circular patch on the crown of the head, having, according to the statements of the mother of the patient, all the characters of ringworm.

Mr. Jabez Hogg, who professes to find parasitic growths in all skin diseases, would, probably, have made use of this case in support of his views, had it come under his observation, whereas a careful examination, and an inquiry in the history of the case, showed it to be a case of ringworm, complicated, in its later stages, with eczema.

*Alopecia areata* (circular patches) of baldness ought never to be mistaken for eczema of the head, and the disease only requires to be kept in view in order to prevent an error in diagnosis. *Favus* is, however, often difficult of distinction from eczema capitis. In cases of favus, "where the head is more or less covered with an eruption exhaling the odor of mice, and consisting of bright yellow dry crusts, depressed in the centre, through the middle of each of which one or more hairs pass, which have a dull, dry appearance, and are more easily extracted than natural, the diagnosis is very easy, and those who have seen the disease once can never mistake it. When it has continued for a length of time, when the crusts have lost their cup-shaped form and their bright yellow color, and have become entangled in the hair; when, in fact, we have to do with the variety described as favus squarrosa, it may be, —and often is—mistaken for impetigo of the scalp. But in the former there are generally patches of alopecia which are wanting in the latter. In it certainly the hairs often fall out, although only here and there, and not in patches as in favus. The alopecia of favus is permanent, that of impetigo generally temporary. There is also no alteration of the hairs in the latter; in the former they are dull, dry, discolored, and easily extracted. Attention to these points generally serves to clear up the diagnosis; but, if doubt still exists, it may at once be removed by the microscopic examination of the crusts. There is one point, however, which requires to be borne in mind, namely, that the discovery of some pustules does not prove that the disease is impetigo, as pustules are frequently developed in cases of favus from the irritation of the parasite. And also one should not lay too great stress on the value, in a diagnostic point of view, of the odor exhaled from the eruption, as this symptom is not so pathognomic as some dermatologists would have us to suppose.

"Very often the diagnosis is rendered difficult on account of a propensity of parents to clean carefully, and remove all the crusts from the head before bringing their children for advice. There is then to be seen redness of the scalp combined with the presence of a few pustules, the results of irritation, and here again the disease resembles impetigo. But if it is a case of favus which we have before us, the deep red, depressed, distinctly circumscribed surface, covered by a thin, shining epidermis, is quite different from the light-colored, diffused redness of impetigo. If this is not sufficient, the hairs should be examined, when they will be found to be altered, and the parasite is detected in them with the microscope. If this is not satisfactory, do not give an opinion or resort to any treatment, but desire the patient to return in a couple of weeks, leaving the head untouched in the *interim*, after which time the disease will have had time to re-develope itself, and its nature is at once discovered.*

In the *treatment* of eczema of the head, the removal of the crusts is often difficult, and the remedial applications cannot be so thoroughly applied to the diseased surface. If the eruption is at all extensive or severe, and if it occurs in children, I am in the habit of ordering the hair to be cut as short as possible, and I always insist upon this, if, as happens too often, particularly amongst the poor, the disease is complicated with lice. The crusts can then be separated with greater facility, and the morbid surface more fully exposed to view. In adults,

* "The Parasitic Affections of the Skin," by T. McCall Anderson, M.D. London: Churchill & Sons. 1861.

and especially in females, the removal of the hair must often be dispensed with, and the cure is, consequently much retarded.

It is only in chronic cases, occurring in adults, and rebellious to milder treatment, that shaving of the head and the application of iodine and blisters (see Lecture in Sept. No.,) is to be recommended; for, in the ordinary run of cases, the milder treatment, before fully discussed, is usually effectual. In very obstinate chronic cases, which resist both internal and external remedies, although very few indeed do not yield to blisters, epilation may be tried, though this is rarely necessary.† If epilation is recommended, and you must remember that it is only to be carried into effect as a "*dernier resort*," you should at the same time use a lotion of bichloride of mercury, or one of the mercurial ointments previously referred to. (See Lecture in Sept. No.)

*Eczema of the Hairy Portions of the Face* (Eczema pilare faciei), is an exceedingly common, and a very annoying affection, owing to the disfigurement which it occasions, the burning heat which accompanies it, and the difficulty and pain of shaving. The only word in English dermatological works which is intended to denote it, is "impetigo menti," but the disease is by no means confined to the chin, so that this name is a too restricted one. I have, therefore, called it "Eczema pilare faciei," which is more correct, though, perhaps, not so euphonious.

The eruption commences by the formation of pustules, each of which is situated at the orifice of a hair follicle; for you will notice that a hair passes through the centre of each pustule. It is curious, though true, that eczema almost always assumes the pustular form in this situation in adult males,—an observation which coincides with what I told you before, that the pustular form of eczema (impetigo) is much more frequently observed on hairy parts of the body.

These pustules dry up into small yellow crusts, which are difficult of removal, owing to their adhesion to the hairs as well as to the skin. When many pustules form at the orifices of neighboring follicles, they have a tendency to run together, and, on drying up, large irregular yellow crusts are left. If these are not removed, successive exudations on the surface of the skin are confined by them, and lead to excoriations, and, owing to their continued pressure, to obliteration of the hair follicles, and permanent alopecia of the affected parts. The

† For the mode of extracting the hairs, see my work on the "Parasitic Affections of the Skin," p. 34. London: Churchill & Sons. 1861.

skin on which the pustules are developed assumes a dusky red tint, and becomes gradually more and more thickened and infiltrated. The patient sometimes complains of itching, oftener of pain or burning heat,—a sensation which is principally experienced during the formation of a crop of pustules. The disease is often kept up for months or even years, owing to the occurrence of successive crops of pustules.

The causes which specially operate in the production of this form of eczema are, irritating discharges from the nose and mouth, and the irritation of the razor, especially when that instrument is blunt. Indeed, the disease not unfrequently disappears spontaneously when the causes are no longer in operation, unless the predisposition to eczema is very strong, or the eruption has lasted a long time.

The diseases with which it may be confounded are acne sycosiformis and sycosis parisitica. In *acne sycosiformis* the eruption partakes more of the tubercular than of the pustular form, and the eruption of acne is detected on the non-hairy parts of the face, and often also between the shoulders and on the front of the chest. Indeed, acne sycosiformis (so named from the resemblance of the eruption to sycosis parasitica,) is merely acne indurata implicating the hairy instead of the non-hairy parts of the face. Strictly speaking, the form of eczema at present under consideration has nearly as much right to be ranked with acne as with eczema, in which case, acne sycosiformis would be regarded as an advanced stage of eczema of the hairy parts of the face. The treatment of the two affections is nearly the same, so that it unnecessary to insist further upon the diagnosis.

The disease which is oftenest confounded with this form of eczema is sycosis parasitica, although the differences are generally very marked. These are well shown in the following table:

| Eczema Pilaris Faciei. | Sycosis Parasitica (Ringworm of the Beard). |
|---|---|
| (1.) Very common in this country. | (1.) Very rare in this country. |
| (2.) A pustular disease only. | (2.) Pustules, tubercles, and large fleshy indurations detected when disease fully established. |
| (3.) No trace of herpes circinatus either on the affected parts or in other localities. | (3.) Rings of herpes circinatus (ringworm of body) detected amongst the hairs, |

| | |
|---|---|
| | and often round the front of the neck, or on the wrists, arms, or other parts of the body. |
| (4.) Not contagious. | (4.) Contagious, and often history of contagion. |
| (5.) Hairs healthy and adhere firmly, so that epilation causes pain, unless much suppuration has occurred at their roots. | (5.) Hairs brittle, broken, and twisted; have lost their natural glistening appearance, and can be extracted with perfect ease and without pain. |
| (6.) No parasite to be detected. | (6.) Fungus (tricophyton tonsurans) detected in the hairs and scales. |

In the cure of this form of eczema, if the means already indicated fail, the patient should be directed to stop shaving, or, if he has a beard, the hair should be cut short. All the hairs proceeding from the affected parts should then be extracted, after which a stimulating and alterative ointment, such as citrine ointment, should be rubbed firmly over the morbid surface night and morning. This treatment often acts like a charm, and I have cured many old standing cases in a couple of weeks by means of it. While it sometimes fails to effect a complete cure, it is always, as far as my experience goes, productive of temporary benefit. After the parts have been once epilated, if new pustules appear, the hair passing through the middle of each must at once be extracted, and the use of the ointment continued.

The following case, and I could cite many such, illustrates the benefit of this mode of treatment. I have selected it because it shows the value of epilation as compared with other treatment:—

Mr. M., aged about 35, consulted me on April 24, 1861, with regard to an eruption on the upper lip, immediately beneath the nostrils. The patch was about an inch square, the skin red and infiltrated, and numerous pustules and yellow crusts were situated at the orifices of the hair follicles. The disease was kept up by the formation of successive crops of pustules. He stated that he had frequently discharge from the nostrils, which he thought irritated the skin of the upper lip. He had been taking Donovan's solution for some time when I saw him, and he said with benefit, and it was, therefore, continued.

A week afterwards, the eruption being in no way altered, Fowler's solution, at first in ten, later in fifteen-drop doses

(thrice daily), was administered for some weeks, and an ointment of two drachms of citrine ointment, mixed with six of linimentum calcis, was rubbed firmly into the roots of the hairs night and morning. The arsenic, in one form or another, having been continued for a couple of months, and pushed till it produced derangement of the digestion, and no benefit accruing from its employment, was omitted, and the morbid surface was touched gently with solid potassa fusa after the removal of the crusts.

A week afterwards, (May 21, 1861,) great improvement was observed. The infiltration and redness of the skin were much less, but still a few pustules continued to form at the edges of the patch.

I now lost sight of the patient till January 23, 1862, when I found the eruption pretty much in the same state as when I first saw him, it having never disappeared entirely. I at once removed the crusts, extracted all the hairs, and ordered citrine ointment to be used night and morning.

Four days later, (January 27, 1862,) the infiltration and redness of the skin were nearly gone, and no new pustules had appeared. He was ordered to continue the use of the ointment a little longer, and, if any new pustules appeared, to pull out the hairs which proceeded through the centres of them.

About two months after this, (March 21,) I saw this gentleman by accident, when he informed me that, since the epilation, the disease had never reappeared, and I could discover no trace of the previous eruption. He wore a magnificent moustache,—epilation, as most of you are aware, having the effect of making the hair grow more luxuriantly than ever, owing to the stimulus which that operation gives to the circulation of the part.

Those of you who are alive to the benefits of a luxurious pair of whiskers, and who have not yet succeeded in the attainment of your wishes, may, perhaps, be inclined to draw a practical lesson from the results of epilation in the case of the gentleman just alluded to.

*Eczema of the Lips* (eczema labiorum) is by no means of rare occurrence, and may coincide with a similar eruption on other parts, though the former are often affected alone. The eruption may be confined to one lip, or both may be implicated, and they may be the seat of any of the forms of eczema previously described. They are often greatly swelled, the serum being diffused through the cellular tissue, the meshes of which are very loose. The oral aperture is often spasmodically contracted, especially if fissures complicate the eruption, as they often do, particularly at the angles of the mouth and the centre of the lower lip.

Hebra has observed eczema of the lips to be frequently associated with eczema of the anus, and he once had a patient who was affected alternately with eczema of the anus and lips.

The two diseases which are most apt to be mistaken for eczema of the lips are, herpes labialis and syphilitic eruptions on these parts. But you will be little likely to fall into error if you remember the points to which I referred in speaking of the diagnosis of eczema in general. There is just one additional circumstance, however, with which you should be familiar in connexion with syphilitic affections of the lips, namely, that the eruption rarely affects the whole of even one lip, but has a marked tendency to concentrate itself at the angles of the mouth, where it is often obstinate till the patient is brought under the influence of mercury, when it, "vanquished, quits the field."

You must be careful in the use of strong solutions of poisonous preparations, such as those of corrosive sublimate, in the treatment of this affection, for it is quite possible for the patient to swallow a sufficiency of the mixture to induce serious symptoms. I have nothing to add with regard to treatment further than to refer you particularly to my remarks upon the means adopted for the removal of limited eczematous eruptions.

The following case of eczema of the lips is a good illustration of the eruption in question:—

"A gentleman, aged about 35, consulted me on April 15, 1861, about an eruption of eczema attacking both lips, and for the second time. A small infiltrated exuding and itching patch existed on the right cheek, near the angle of the mouth, and occasionally vesicles were detected on it. The lips were slightly infiltrated, thickened, red, and itching; the epithelium was constantly peeling off them, so that they were very rough, and sometimes a little serous fluid exuded, while fissures had formed here and there, but particularly at the angles of the mouth. His general health was excellent. Fowler's and Donovan's solutions were successively administered without effect, and the disease was finally and rapidly cured by applying "aqua potassa" to the parts night and morning, and washing them frequently with cold water."

*Eczema of the edges of the Eyelids* (*Eczema tarsi, Ophthalmia tarsi, Tinea ciliorum,*) is exceedingly common, especially in scrofulous children, and in them often associated with conjunctivitis and strumous ophthalmia. The affection is neither more nor less than a pustular eczema (impetigo), attacking the edges of the lids, although is does not seem to be generally recognized as such by ophthalmic surgeons; for it commences by the formation of pustules at the orifices of the hair-follicles, which concrete into scabs, beneath which small ulcers are detected, and, when the disease is fully developed, the usual symptoms of eczema, itching, infiltration, exudation, etc., are observed. The exudation from the morbid surface, mingled with the altered secretion from the Meibomian follicles, glues the edges of the eyelids together, especially at night, unless proper precautions are taken. Lachrymation is likewise a common symptom, and the tears, falling on the cheek, not unfrequently irritate the skin, and give rise to an eczematous eruption.

If improperly treated or neglected, as occurs too often, the pressure of the crusts, the confinement of the discharge, and the formation and extension of ulcers, lead ultimately to obliteration of the Meibomian glands and hair-follicles, after which, a perfect cure is of course impossible. Amongst the train of evils may also be mentioned eversion or inversion of the lids, and, if the eyelashes are not gone, owing to obliteration of their follicles, the hairs are apt to assume abnormal directions.

With regard to the local treatment, (for the constitutional, see the treatment of eczema generally), the extraction of the eyelashes is always followed by improvement. This operation is far too often omitted, for, in my opinion, it should be uniformly carried into effect in bad cases, and repeated if new pustules form at the orifices of the follicles, exactly in the same way as in the treatment of eczema of hairy parts of the face. (See treatment of eczema of hairy portions of face.) In addition to this, if the parts are much infiltrated, I am in the habit, after the removal of all crusts, of applying a solution of potassa fusa (usually a solution of ten grains in an ounce of water,) to the edges of the lids, an operation which should not be entrusted to the patient, at first at all events. A small brush must be used, and very little of the solution taken up by it, so as to make it moist, but no more. The eyelid must then be carefully dried, else the application spreads, everted so as to remove it from the eyeball, and the solution painted along its edge. A large brush soaked in cold water should be in readiness to stop the action when desired. This application may be repeated every day till the infiltration, exudation, and itching subside, after which citrine ointment may be relied upon for completing the cure. In slight cases, you will often find the eruption yield to the use of citrine ointment alone, coupled with cleanliness. During the treatment of eczema tarsi, a little ointment (diluted citrine ointment, for example,) should be applied to the edges of the lids at night, so as to prevent their adhesion, but it must always be washed off in the morning. If, notwithstanding the anointing of the lids at night, they are adherent in the morning, you must on no account tear them asunder, but must previously soften the agglutinated matter. Towards this end you cannot go wrong if you follow the instructions of Mackenzie:—"A teaspoonful of milk, with a bit of fresh butter melted in it, may be employed for smearing the lids, rubbing it with the finger gently along the agglutinated eyelashes. A piece of soft sponge, wrung out of hot water, is then to be held upon the eyelids for some minutes, after which the patient will find the eyelids yield without pain to the least effort he makes to open them. With the finger-nail the whole of the matter is immediately to be removed."*

If there is any inflammation of the conjunctiva, Mackenzie's excellent wash of the bichloride of mercury may be used with advantage, and is often sufficient when the conjunctivitis is slight.

℞. Hydrargyri Bichloridi,.................. gr. j.
Hydrochloratis Ammoniæ,............ gr. vj.
Cocci Cacti,.............................. gr. jss.
Alcoholis,.................................. ʒj.

Tere simul, adde aquæ uncias sex, et cola per chartam.

*Siqua.*—Pour out half a tablespoonful of this fluid, and mix it with as much boiling water in a teacup previously warmed. With a piece of old linen or soft sponge bathe the eyelids with the mixture for a few minutes, and then, by leaning back the head, allow a little of it to flow in upon the eye. Repeat this thrice daily.

For the treatment of a more severe attack, as well as for that of the other complications of eczema tarsi, such as ectropium, entropium, trichiasis, ophthalmia scrofulosa, etc., I must refer you to special works on ophthalmic surgery.

* "A Practical Treatise on the Diseases of the Eye," by W. Mackenzie, M.D. Fourth Edition, p. 145. London: Longman, Brown, Green, and Longmans.

